# 4448 Mental Stress Induced Myocardial Ischemia as a Marker for Adverse Cardiovascular Events After MI

**DOI:** 10.1017/cts.2020.134

**Published:** 2020-07-29

**Authors:** Zakaria Almuwaqqat, Bruno Lima, An Young, Samaah Sullivan, Amit Shah, Muhammad Hammadah, Ernest Garcia, Douglas Bremner, Paolo Raggi, Arshed Quyyumi, Viola Vaccarino

**Affiliations:** 1 Emory University

## Abstract

OBJECTIVES/GOALS: Young and middle-aged adults with a myocardial infarction (MI) represent an understudied group potentially with unique risk indicators such as emotional stress. We sought to investigate if mental stress-induced myocardial ischemia (MSIMI), a marker of cardiovascular vulnerability to psychological stress, is associated with poor outcomes among this population. METHODS/STUDY POPULATION: We studied 306 patients (150 women and 156 men) ≤61 years of age who were hospitalized for MI in the previous 8 months. Clinical, behavioral and psychosocial factors were assessed with standardized measures. Patients underwent myocardial perfusion imaging with mental stress (public speaking) and conventional stress (exercise or pharmacological testing). MSIMI and conventional stress-induced ischemia were defined as a new or worsening perfusion defect. Patients were followed for 3 years for adverse events, which were independently adjudicated. Cox proportional hazard models were used to estimate the association of MSIMI and CSIMI with a composite endpoint of recurrent MI or cardiovascular (CV) death with adjustment for demographic, clinical and psychosocial risk factors. RESULTS/ANTICIPATED RESULTS: The mean age of the sample was 50 years (range, 22-61). MSIMI occurred in 16% of the patients, and conventional ischemia in 35%. Over a 3-year follow-up, 28 individuals had a recurrent MI and 2 died due to cardiovascular causes. The incidence of the composite endpoint of MI or CV death was more than doubled in patients with MSIMI (20%) than those without MSIMI (8%), HR 2.6, 95%CI, 1.2-5.6. Further adjustment for demographic and clinical risk factors and depressive symptoms did not substantially change the relationship. In contrast, conventional stress ischemia was not significantly related to the outcome (HR 1.4, 95%CI, 0.6-3.0). DISCUSSION/SIGNIFICANCE OF IMPACT: Young and middle-aged individuals with MSIMI after MI have a >2-fold higher likelihood of recurrent MI and CV mortality compared with those without MSIMI. In this patient group, MSIMI is a better risk indicator than ischemia with a conventional stress. These findings point to psychological stress as an important determinant of risk in this patient population. Ischemia induced by mental stress is a potent risk indicator in young post-MI patients. Stress-reduction interventions may be especially beneficial in patients who show this abnormal response.

